# Impact of Reconstitution Conditions on the Solubility of Faba Bean Protein Isolate

**DOI:** 10.3390/foods13233857

**Published:** 2024-11-29

**Authors:** Rui Yu, Thom Huppertz, Todor Vasiljevic

**Affiliations:** 1 Advanced Food Systems Research Unit, Institute for Sustainable Industries and Liveable Cities, College of Sports, Health and Engineering, Victoria University, Melbourne 8001, Australia; rui.yu3@live.vu.edu.au (R.Y.); thom.huppertz@frieslandcampina.com (T.H.); 2 FrieslandCampina, 3818 LE Amersfoort, The Netherlands; 3 University College Cork, T12 K8AF Cork, Ireland

**Keywords:** faba bean protein isolate, solubility, secondary structure, temperature, pH, water hardness, globulin

## Abstract

Faba bean protein isolate (FBPI) is emerging as a promising protein ingredient in the food industry. However, a lack of comprehensive scientific understanding of its functional properties, particularly solubility, limits broader application. This study investigated the reconstitution behaviour of FBPI under different conditions. For this purpose, FBPI dispersions (5% *w*/*w* protein) were prepared with varying pH (6.8 or 7.5), temperature (15, 40, or 65 °C), duration of stirring (30, 60, or 90 min), stirring intensity (1000 or 1500 rpm), and water hardness (0, 200, or 400 ppm). Low reconstitution temperature resulted in greater particle size and lower solubility, while elevated temperature minimised intermolecular attractions, improving solubility. Higher pH increased the net-negative charge and thus enhanced the repulsion between the proteins, leading to greater solubility. Water hardness was another important parameter, as greater hardness generally resulted in greater particle size and lower solubility, likely due to calcium bridging. The selection of conditions for the hydration of faba bean protein isolate is important to produce high-quality and high-stability suspensions.

## 1. Introduction

The world population is expected to reach almost 10 billion by 2050 [1]. This growing population places additional pressure on existing food resources. A transition towards an increased proportion of plant-derived dietary substances has been suggested to allow for sustainable and prospective provision of proteins [2,3]. Nutritional plant resources can offer solutions for the future food supply. Pulses, e.g., peas, chickpeas, lentils, and faba beans, represent a significant dietary protein source [4]. They are rich in lysine, leucine, aspartic acid, glutamic acid, and arginine; pulse proteins can contribute to a well-balanced essential amino acid profile, particularly when combined with cereals and other sulphur-containing amino acid and tryptophan-rich protein sources [5]. Next to their nutritional value, pulse proteins exhibit notable functional properties crucial for food formulation and processing. These functional attributes include solubility, water-binding and emulsification, and foaming.

Faba beans (*Vicia faba* L.) are among of the most widely grown grain legumes [6]. Their production can lower greenhouse gas emissions compared to wheat monoculture and increase soil nitrogen fixation [7]. Faba beans also provide proteins and other valuable nutrients, as well as dietary fibre and bioactive compounds [8,9]. Faba beans have extensive historical utilisation as a dietary source for human consumption, with archaeological evidence tracing the oldest seeds of faba beans back to the late 10th millennium B.P. in north-west Syria [10]. Faba beans contain nearly twice the protein content compared to cereal grains, and the protein fraction is composed of four protein classes, i.e., globulins (55–60%), albumins (20%), glutelin (15%), and prolamins (8%) [11]. Faba bean globulins represent 70–80% of the storage proteins in faba beans and are usually classified into two classes based on their sedimentation coefficient: the 7S vicilin-type and 11S legumin-type globulins [12].

The solubility of plant proteins plays an important role in determining the quality attributes of foods [13]. The utilisation of faba bean proteins in foods for human consumption remains limited due to their low solubility and sub-par functionalities compared to animal proteins [14]. Previous research demonstrated that the extraction process greatly affected the functional properties of faba bean proteins and may lead to protein denaturation and aggregation [15]. In addition, faba bean proteins in solution tend to generate large insoluble aggregates primarily attributable to the dense configuration of storage proteins [16]. These proteins aggregate under alkaline conditions (pH 10–12) employed during the initial extraction of faba bean proteins.

The intermolecular interactions between faba bean proteins under industrially relevant processing conditions, including pH, temperature, and treatment duration, were reflected by protein activities such as the aggregation and hydrolysis of faba bean proteins [17]. The solubility of proteins typically increases with temperature up to approximately 50 °C, above which proteins may undergo destabilisation of the secondary and tertiary structure, attributable to the disruption of non-covalent bonds. Elevated temperatures commonly give rise to inter-protein interactions, leading to diminished solubility due to aggregation and precipitation [18,19,20]. Moreover, the presence of hydrophobic patches on protein surfaces may impair solubility. In contrast, favourable solubility is associated with a charged protein surface, implying that the ionic environment may play a role [21,22,23,24]. Previous studies identified the iso-electric point of faba bean protein isolate (FBPI) at pH 4.5 [25]. Therefore, any hydration of FBPI should be done at a pH away from its isoelectric point, either at a neutral or even higher pH [21].

Additionally, water hardness, defined as the amount of dissolved calcium and magnesium in water, is a critical parameter for food quality, as water serves as the primary solvent in food processing [26]. Its quality can significantly influence the final product’s solubility, stability, and nutritional value. During reconstitution processes, powders are typically dispersed in water, using shear forces, which have the potential to induce both reversible and irreversible changes in the conformation of proteins, including unfolding and aggregation [27,28,29].

This study aimed to establish the impact of selected commercially applicable conditions relevant to FBPI hydration. These conditions included temperature, pH, water hardness, stirring rate, and mixing time, collectively as diverse parameters. All of these factors impact protein solubility, as they impact all other protein functionalities, such as emulsification, foaming, and gelation [8]. To establish the effect of commercially relevant processing conditions for the faba bean protein, pH 6.8 and 7.5 were selected, since many plant protein beverages were prepared at these pH levels. The mixing and duration rate were also based on the commercial applications. Hence, it is imperative to consider all of these factors to identify the optimal rehydration conditions for achieving maximum solubility.

## 2. Materials and Methods

### 2.1. Material

FBPI powder with a protein content of 88% (*w*/*w*, dry basis) was kindly donated by Australian Plant Proteins Pty Ltd. (West Melbourne, VIC, Australia). Unless otherwise mentioned, all the chemicals used in this study were analytical and obtained from Sigma-Aldrich Pty Ltd. (Castle Hill, NSW, Australia). Milli Q water (Merck Millipore, Bayswater, VIC, Australia) was used throughout the experiment.

### 2.2. Sample Preparation

Approximately 780 mL of water with different WH, adjusted by the addition of 0, 200, or 400 ppm of CaCO_3_ and pH (6.8 or 7.5), was poured into a beaker and adjusted to 15, 40, or 65 °C by holding in a water bath. Once the temperature reached the required level, FBPI powder was poured into the beaker under constant stirring (1000 or 1500 rpm) using an overhead stirrer (ISG, Westlab PTY Ltd., Ballarat, Australia). The temperature was allowed to stabilise, and then pH was adjusted to 6.8 or 7.5 using 1 M NaOH or 1 M HCl if required. Finally, the sample volume was adjusted to 800 mL, and the sample was stirred at 1000 or 1500 rpm for 30, 60, or 90 min.

### 2.3. Determination of Protein Solubility

Upon completing the sample rehydration procedure, the solubility of the protein in all samples was established by centrifugation (Model J2HS; Beckman, Fullerton, CA, USA) at 700 g and 20 °C for 10 min. The solubility was calculated as described previously [30] as the protein content in the supernatant relative to that of the original dispersion:

% Solubility = (Protein content of supernatant)/(Protein content of the original dispersion) × 100

The protein content was determined using the Kjeldahl method [30].

### 2.4. Zeta Potential and Particle Size

Zeta potential and average particle size were determined using a Zetasizer Nano S (model ZS, Malvern Instruments Ltd., Malvern, UK) at 20 °C. All samples were diluted 1:100 with Milli Q water, and measurements were conducted at 20 °C. The refractive index for particles and the dispersant (water) were set at 1.45 and 1.33, respectively. All samples were analysed at least three times.

### 2.5. Attenuated Total Reflectance Fourier Transform Infrared Spectroscopic (ATR-FTIR) Analysis of FBPI Dispersions

Immediately after reconstitution, both bulk and supernatant of 5% (*w*/*w*) protein dispersion samples were analysed by an ATR-FTIR (Frontier 1, PerkinElmer, Waltham, MA, USA) in the range of 4000–600 cm^−1^ at a resolution of 4 cm^−1^ and averaging 16 scans for each spectrum for evaluating the protein conformation. FTIR spectra were obtained at room temperature (~20 °C) shortly after each treatment. The ATR-FTIR was equipped with an ATR cell that allows for the measurement of liquid samples. To discern specific secondary structures for selected samples, the second derivative of all spectra in the broad Amide I region (1700–1600 cm^−1^) was calculated using Spekwin32-Spectragryph software (version 1.2.16). Peaks observed at different wavenumbers were assigned to specific secondary structures: intermolecular β-sheets (IEM β-S)—1612–1630 cm^−1^, β-sheet elements (1630–1648 cm^−1^), α-helix (α-H; 1649–1658 cm^−1^), β turns (βT; 1659–1675 cm^−1^), intramolecular β-sheets (IAM β-S; 1676–1690 cm^−1^), and aggregated β-sheets (A β-S; 1690–1699 cm^−1^) [31,32,33,34]. To calculate the proportion of the structural elements for selected samples, the second derivative and curve-fitting procedures were employed to locate overlapping peaks in the Amide I region using Origin Pro 2024 software (v10.15, Origin Lab Corporation, Northampton, MA, USA) [34,35]. The relative proportions of different secondary structures of FBPI were determined by computing areas of spectral components (Gaussian peaks) assigned to a particular substructure in the Amide I region [22].

**Table 1:** The proportion of different secondary structural elements in the Amide I region of faba bean protein isolate dispersions subjected to different temperatures (15 or 65 °C) and stirring (1000 or 1500 rpm).

Temperature (°C)	pH	Water Hardness (ppm)	Stirring Rate (rpm)	Time (min)	Intermolecular β-Sheet Aggregates	β-Sheet Elements	α-Helix	β-Turns	Intermolecular β-Sheets	Aggregated β-Sheets
					(1620–1630 cm^−1^)	(1630–1645)	(1646–1660)	(1660–1675)	(1680–1690)	(1690–1700)
15	6.8	400	1000	30	8.69 ± 0.93 ^AB^	14.56 ± 1.00 ^AB^	13.27 ± 2.69 ^B^	13.14 ± 2.07 ^B^	11.98 ± 1.71 ^A^	8.39 ± 1.41 ^BC^
				60	7.54 ± 1.78 ^AB^	13.21 ± 2.45 ^AB^	14.97 ± 2.33 ^B^	12.73 ± 0.94 ^B^	12.26 ± 1.39 ^A^	11.92 ± 1.27 ^A^
				90	8.10 ± 2.05 ^AB^	12.96 ± 2.24 ^AB^	13.33 ± 1.15 ^B^	13.62 ± 1.51 ^B^	12.19 ± 1.45 ^A^	13.48 ± 0.35 ^A^
65	7.5	0	1500	30	10.29 ± 0.74 ^A^	17.28 ± 0.74 ^AB^	17.54 ± 0.21 ^A^	14.25 ± 0.25 ^B^	8.98 ± 0.70 ^A^	5.05 ± 0.23 ^C^
				60	2.10 ± 0.04 ^C^	12.66 ± 1.70 ^B^	23.07 ± 3.92 ^A^	20.32 ± 1.80 ^A^	12.00 ± 1.39 ^A^	8.80 ± 0.89 ^B^
				90	5.18 ± 2.05 ^BC^	17.80 ± 1.96 ^A^	16.04 ± 0.36 ^B^	18.28 ± 3.77 ^AB^	11.34 ± 0.22 ^A^	6.20 ± 0.58 ^C^

Values are mean ± SD. Means with the same superscripts in a column did not differ significantly (*p* > 0.05).

### 2.6. Sodium Dodecyl Sulphate Polyacrylamide Gel Electrophoresis (SDS-PAGE)

The samples with lowest and highest solubility were selected for analysis using SDS-PAGE under reducing and non-reducing conditions [34]. Following the mentioned treatment, both bulk and supernatant of FBPI samples were combined with SDS-PAGE sample buffer and subsequently subjected to electrophoresis. They were prepared by diluting a 5% (*w*/*w*) protein dispersion to a final concentration of 1.0 mg/mL. β-Mercaptoethanol was used as the reducing agent, while broad-range pre-stained SDS-PAGE standards (SeeBlue Plus2 Pre-Stained Protein Standard, Thermo Fisher Scientific, Scoresby, VIC, Australia) were used as molecular weight markers. After staining and destaining, gel images were captured using a ChemiDoc imager (Chemidoc MP, Bio-Rad Laboratories, Hercules, CA, USA).

### 2.7. Statistical Analysis

The experiments were planned as a randomised, blocked, split-plot design with temperature as the main plot, and pH, water hardness (WH), stirring intensity, and stirring time as subplots. This block was replicated to obtain at least four independent observations (n ≥ 3). The results were analysed using the GLM procedure of SAS (v.9.2) to determine either individual or combined effects of the factors. The level of significance was pre-set at *p* ≤ 0.05. The analyses were performed in triplicate. Data were tested for normality, and results were analysed using a one-way analysis of variance (ANOVA) followed by Tukey’s post hoc test (*p* < 0.05).

## 3. Results

### 3.1. Solubility of Faba Bean Protein Dispersions

**Figure 1:**
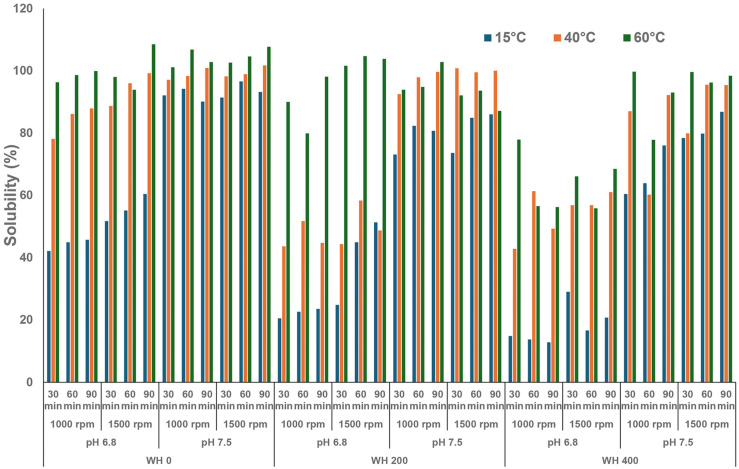
Solubility of FBPI dispersions reconstituted under various solution conditions (pH—6.8/7.5, water hardness—0, 200, or 400 ppm, shearing rate—1000 or 1500 rpm, time—30, 60, or 90 min) at 15, 40, or 65 °C.

In this study, the reconstitution of FBPI was affected by temperature, pH, and WH. Temperature played the greatest role in solubility among these parameters. The lowest solubility was observed at 15 °C; once the temperature increased to 40 °C, the lowest solubility at this temperature level was three times greater than that at 15 °C (Figure 1). Additionally, most protein dispersions were characterised by solubility of over 90% at 65 °C. At 15 °C, solubility was notably affected by pH and WH. Though the solubility can be decreased with the rising WH level or the pH decrease, the disparity in solubility between different pH and WH was diminished at elevated temperatures. FBPI solubility at pH 6.8 and 7.5 was over 90% at 65 °C and at a WH of 0 ppm (Figure 1).

The solubility at pH 7.5 was greater than at pH 6.8 at each WH and temperature (Figure 1). With increasing WH from 0 to 400 ppm at 15 °C and pH 6.8, there was a 50% reduction in solubility (Figure 1). In contrast, at pH 7.5, the decrease in solubility with increasing WH was smaller than that at pH 6.8. Apart from the positive effect of increasing temperature and pH, the higher WH decreased the solubility of FBPI, so the lowest solubility was observed at a WH of 400 ppm (Figure 1).

### 3.2. Zeta Potential and Particle Size

**Figure 2:**
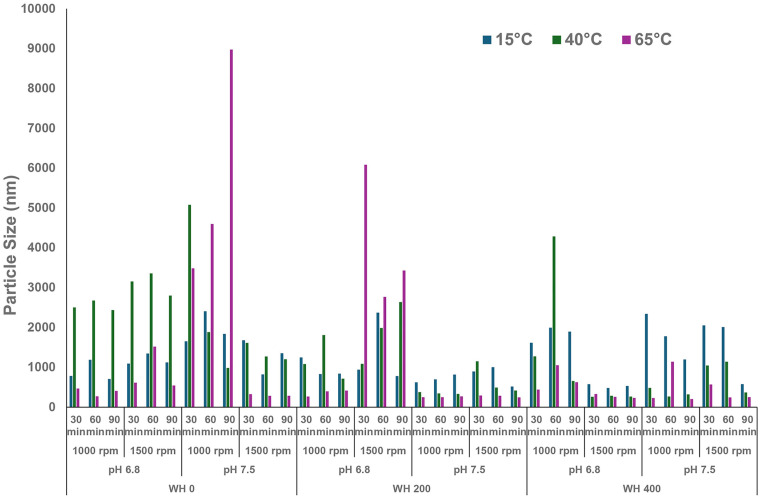
Particle size of FBPI dispersions reconstituted under various solution conditions (pH—6.8/7.5, water hardness—0, 200, or 400 ppm, shearing rate—1000 or 1500 rpm, time—30, 60, or 90 min) at 15, 40, or 65 °C.

The particle size of FBPI dispersions was primarily influenced by temperature, pH, and WH (Figure 2). A decrease in the particle size of the bulk and the supernatant was observed with a rise in temperature. The difference in particle size between the bulk and the supernatant decreased as the reconstitution temperature increased. The particle size of both the bulk and supernatant of the dispersions was larger at pH 6.8 than at pH 7.5 at all temperatures. Furthermore, particle size increased at each temperature as WH increased.

**Figure 3:**
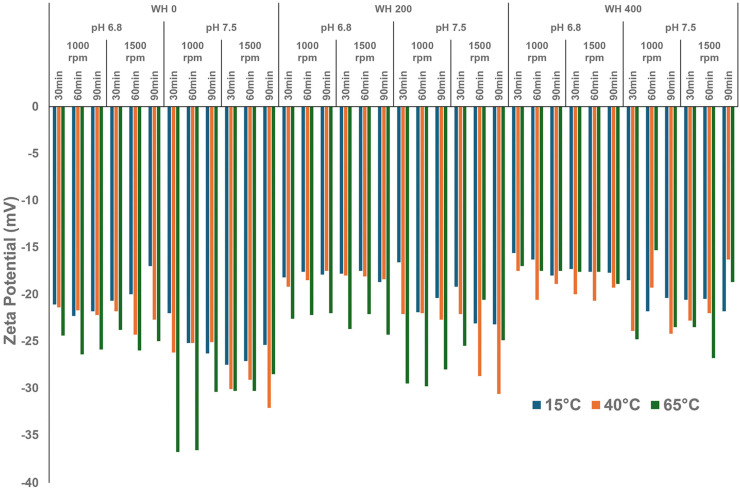
Zeta potential of FBPI dispersions reconstituted under various solution conditions (pH—6.8/7.5, water hardness—0, 200, or 400 ppm, shearing rate—1000 or 1500 rpm, time—30, 60, or 90 min) at 15, 40, or 65 °C.

Temperature, pH, and WH also affected the zeta potential of FBPI dispersions (Figure 3). As the temperature, pH, and water hardness increased, the zeta potential changed from −15.6 to −34.2 mV. At pH 7.5, the negative zeta potential was greater than at pH 6.8. At 15 °C, the zeta potential at pH 6.8 and 7.5 were similar, mostly between −16 and −22 mV. In contrast, when reconstitution was done at 65 °C, most dispersions had a zeta potential lower than −22 mV. It was characterised by an initial increase followed by a subsequent reduction. This study observed the highest net-negative charge at a WH of 200 ppm of approximately −34 mV (Figure 3).

### 3.3. Changes in the Secondary Structure of Proteins as Impacted by Reconstitution Conditions

**Figure 4:**
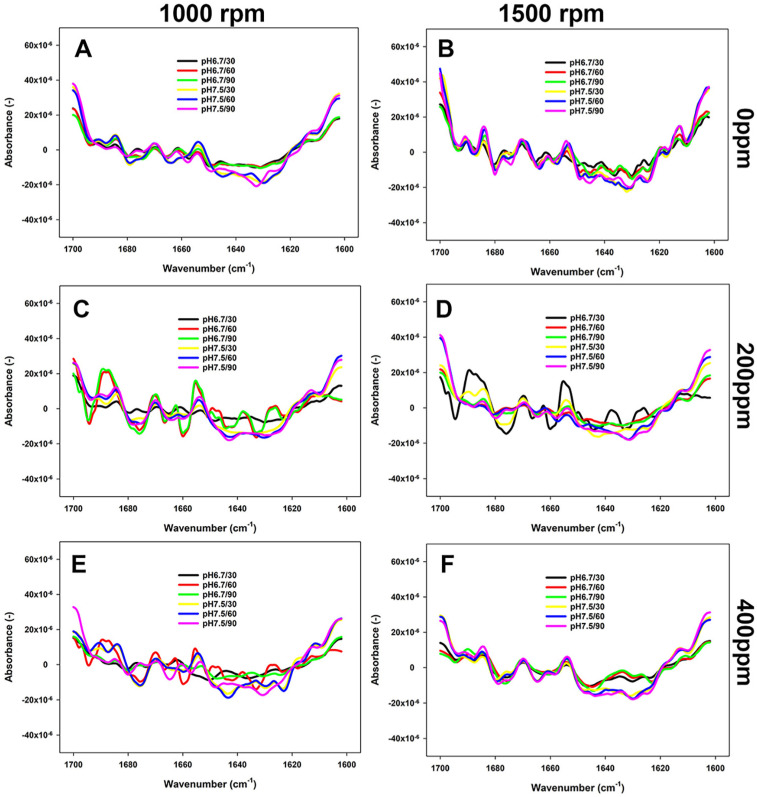
FTIR spectra (second derivative) of 5% FBPI dispersions held under different pH (6.8 or 7.5) and water hardness (0, 200, or 400 ppm) and sheared at 1000 rpm or 1500 rpm at 15 °C. (**A**,**C**,**E**) refer to dispersions prepared by stirring at 1000 rpm in water with a hardness of 0, 200 or 400 ppm; (**B**,**D**,**F**) refer to dispersions prepared by stirring at 1500 in a water with a hardness of 0, 200 or 400 ppm.

**Figure 5:**
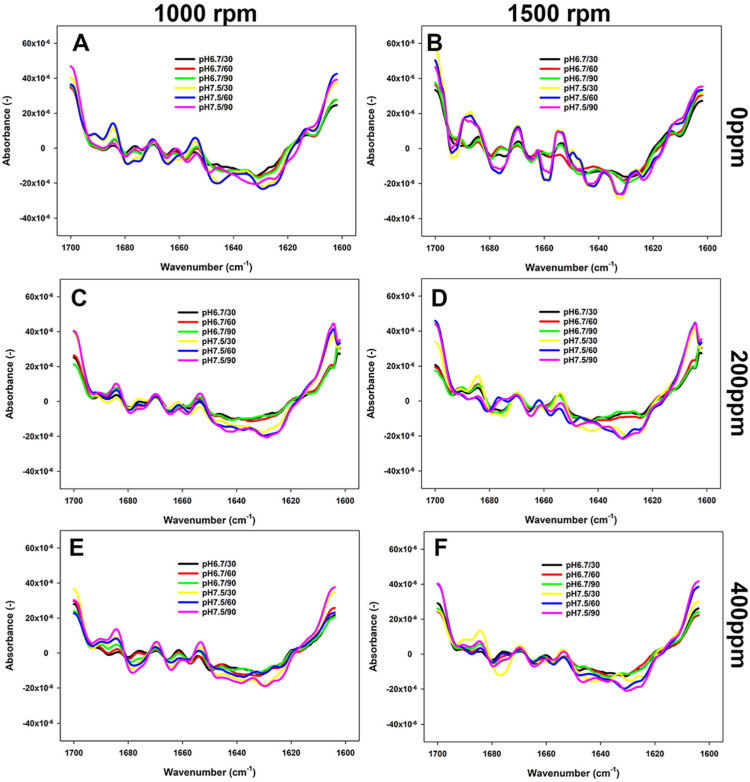
FTIR spectra (second derivative) of 5% FBPI dispersions held under different pH (6.8 or 7.5) and water hardness (0, 200, or 400 ppm) and sheared at 1000 rpm or 1500 rpm at 40 °C. (**A**,**C**,**E**) refer to dispersions prepared by stirring at 1000 rpm in water with a hardness of 0, 200 or 400 ppm; (**B**,**D**,**F**) to dispersions prepared by stirring at 1500 in a water with a hardness of 0, 200 or 400 ppm.

**Figure 6:**
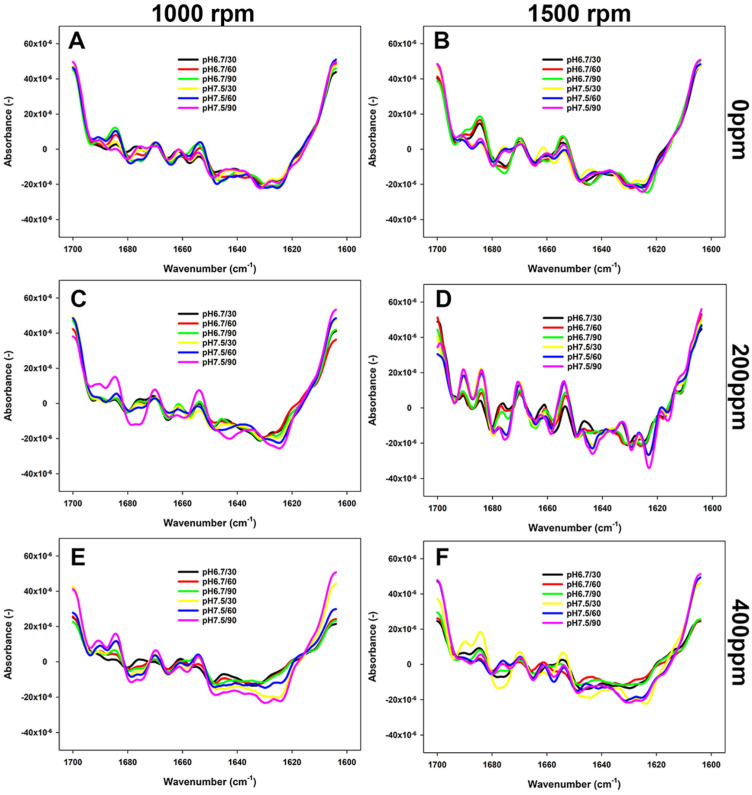
FTIR spectra (second derivative) of 5% FBPI dispersions held under different pH (6.8 or 7.5) and water hardness (0, 200, or 400 ppm) and sheared at 1000 rpm or 1500 rpm at 65 °C. (**A**,**C**,**E**) refer to dispersions prepared by stirring at 1000 rpm in water with a hardness of 0, 200 or 400 ppm; (**B**,**D**,**F**) to dispersions prepared by stirring at 1500 in a water with a hardness of 0, 200 or 400 ppm.

The changes in the secondary structure of proteins in FBPI dispersions were studied by FTIR. As shown in Figure 4, Figure 5 and Figure 6, the most structural changes were observed in the FBPI dispersions at pH 6.8 and water hardness 200 ppm under each temperature level. These changes appear to be associated with a reduction of major secondary structures such as α-helix (~1655 cm^−1^), β-sheet (~1638 cm^−1^), β-turn (~1670 cm^−1^), and anti-parallel β-sheet (~1689 cm^−1^) subjected to varying pH and water hardness.

According to Figure 4, the prevalence of β-sheet, α-helix, and anti-parallel β-sheets structures was greater in samples at 400 ppm WH and pH 7.5 than those observed at pH 6.8 and WH 0 ppm at 15 °C. At 40 °C, the peaks of anti-parallel β-sheet, α-helix, and β-turn structures at pH 7.5 were more noticeable than those at pH 6.8 at each WH (Figure 5). Consequently, as the temperature increased from 40 to 65 °C at a WH of 200 or 400 ppm, the peaks became even more pronounced, particularly at 1500 rpm (Figure 6). At this temperature and 0 ppm WH, the peaks remained unchanged regardless of the change in pH or shear rate (Figure 4, Figure 5 and Figure 6).

To examine these conformational fluctuations more closely, changes in the proportion of relevant structural elements in samples with the greatest and poorest solubility are shown in Table 1. The parameters defining the low solubility group were as follows: a temperature of 15 °C, a pH of 6.8, a WH of 400 ppm, and an agitation speed of 1000 rpm, reconstituted for 30, 60, and 90 min. The high-solubility group was characterised by an elevated temperature of 65 °C, pH of 7.5, and an agitation speed of 1500 rpm, combined with a water hardness of 0 ppm. As indicated, the poorer solubility of FBPI appeared to be associated with the loss of α-helical structure and β-turns and the rise in intermolecular β-sheet aggregates and aggregated β-sheets. Conversely, the increasing proportion of α-helices and β-turns at pH 7.5 appeared to align with greater solubility of FBPI (Table 1). More specifically, the mean relative proportion of α-helix and β-turn increased from 13.3% and 13.6% to 16.0% and 18.3%, respectively, when the duration of stirring was 90 min. At the same time, these soluble dispersions were characterised by the lowest proportion of intermolecular β-sheet aggregates and aggregated β-sheets (Table 1). Interestingly, the proportion of intermolecular β-sheet aggregates in high-solubility dispersions was fairly high (10.3%) after 30 min of stirring; however, it significantly decreased to 2–5% after 60 or 90 min of stirring (Table 1).

### 3.4. Protein Partitioning

**Figure 7:**
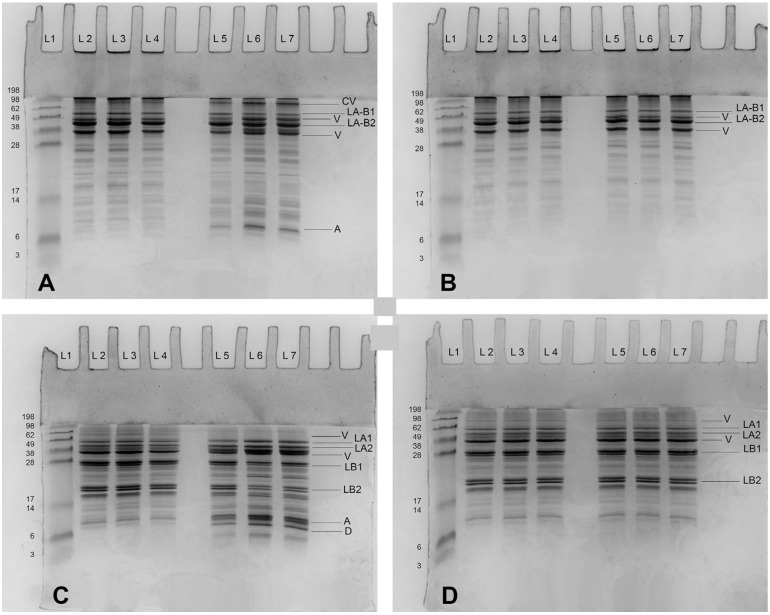
Non-reducing (NR) and reducing (R) SDS-PAGE analysis of 5% (*w*/*w*) FBPI dispersions (bulk) and supernatant at 15 °C, pH 6.8, WH (water hardness) 400 ppm, 1500 rpm (**A**), 65 °C, pH 7.5, WH 0 ppm, 1500 rpm (**B**), reducing WH 400 ppm, 1500 rpm (**C**), and 65 °C, pH 7.5, WH 0 ppm, 1500 rpm (**D**). Lanes are designated as L1 molecular weight markers; L2 bulk sample 30 min, L3 bulk sample 60 min, L4 bulk sample 90 min, L5 supernatant mixing time 30 min; L6 supernatant mixing time 60 min; L7 supernatant mixing time 90 min.

Non-reducing SDS-PAGE analysis revealed changes in the soluble proteins’ type and molecular weight distribution in the different suspensions. Figure 7 illustrates that faba proteins comprised several components primarily identified as legumin and vicilin. When non-reducing and reducing SDS-PAGE were compared, notable differences could be attributed to bands at approximately 56 and 46 kDa, corresponding to legumin A-B 1 and A-B 2, respectively (Figure 7). Legumin comprises six subunit pairs, each linked by a single disulphide bond [36]. According to Figure 7, two types of legum7in, namely A and B, and two acidic and basic (AB) subunits could be identified under non- and reducing SDS-PAGE conditions. Under reducing conditions, three large protein complex bands at the top of the well disappeared, accompanied by the appearance of four new intense bands at 44, 34, 26, and 20 kDa (Figure 7), which were previously identified as α-legumin 1, α-legumin 2, β-legumin 1, and β-legumin 2, respectively [36].

Similar to the FTIR analysis, two groups of samples were again selected for further investigation based on their solubility. In the low solubility group, less intense legumin bands indicated that less legumin protein remained dispersed in the supernatant phase (Figure 7A). At the same time, no protein aggregates remained at the top of the PAGE well (Figure 7A). In contrast, a more significant proportion of aggregates at the top of the well for both bulk and supernatant samples was observed at higher temperatures (Figure 7B). Additionally, the increased intensity in bands of legumin subunits aligned with the increasing solubility (Figure 7B). Therefore, the molecular behaviour of legumin appears to play a crucial role in the functionality of the faba bean protein isolate.

In addition, the bands at 51, 45–47, and 18 kDa have been previously identified as vicilin subunits [25]. As vicilin contains no disulphide bonds in its structure, these subunits were consistently present in both reducing and non-reducing SDS-PAGE. Additionally, the convicilin subunits (70 kDa) could be identified at low temperatures and present at a greater concentration (Figure 7A) while disappearing at higher temperatures, likely by centrifugation-induced precipitation, which may indicate that they were held by hydrophobic attraction.

Assessment of the supernatant composition revealed that the supernatant at low temperature contained many bands of a relatively low molecular weight, ranging from 6 kDa to around 28 kDa. Conversely, most proteins in the supernatant at 65 °C appeared larger and distributed around 34–198 kDa or aggregated in the loading well (Figure 7B). Differences appeared even more notable when the protein aggregates were reduced (Figure 7D). Specifically, albumins eluted at 10–12 kDa, showing only the lowest solubility group in this study (Figure 7A) [37]. They are usually present after the short processing time, and interact with other charged clusters via electrostatic attractions during the prolonged shearing process, creating aggregates [38]. Another small band observed in Figure 7C can be regarded as a defensin-like protein (9.5 kDa), only visible at low temperatures [37]. As it is sensitive to temperature, the defensin-like protein was not apparent in the PAGE obtained of the samples at high temperatures (Figure 7B).

## 4. Discussion

Faba bean proteins comprise four protein classes: globulins, albumins, glutelins, and prolamins [11]. Globulins, due to their abundance, play an essential role in the functional performance of FBPI under various processing conditions. They are categorized based on their sedimentation coefficients into two primary types: legumin (11S) and vicilin (7S) [39]. Legumins are the predominant globulins in faba beans, accounting for up to 55% of the seed proteins [40]. They exist in hexameric structures (320–400 kDa), with each subunit consisting of acidic (α) and basic (β) peptide chains linked by disulphide bonds [41]. On the other hand, vicilin exists in a trimeric structure (145–190 kDa) with non-identical subunits [18]. They are glycosylated and lack cysteine, which prevents them from forming disulphide bonds. The third globulin of faba bean protein is convicilin, with a MW of 52–99 kDa, which was regarded as belonging to vicilin [37,42,43]. More specifically, the typical protein profile of faba bean consists of legumin minor subunit (80 kDa), convicilin subunit (70 kDa), legumin major subunit (44–34 kDa), vicilin subunit (51–18 kDa), and albumin (10–20 kDa) [18,25,36], as it was confirmed in the current study (Figure 7).

Reconstitution conditions greatly impact the structural features of proteins and, thus, their reactivity. In this sense, this study focused on assessing the changes in the Amide I region to understand how the selected processing conditions impacted the secondary structure backbone conformation (Figure 4, Figure 5 and Figure 6). Modifications in the conditions of the dissolved protein can affect its structural motifs, including intermolecular β-sheet aggregates and aggregated β-sheets, and α-helix and β-turns (Table 1). The 7S and 11S proteins have a substantial proportion of pleated β-sheet motifs, comprising around 30% of these proteins, in addition to intra- and intermolecular β-sheets, which together account for about 40% [33,44,45,46]. Eckert et al. [15] reported that β-sheets were the predominant secondary structural element in a faba bean protein extract. Our study has also confirmed these findings, as β-sheets were one of the main characterised conformational elements. In contrast, the contribution of the α-helix structure has been reported to be around 10% in the FBPI [46], similar to what was observed in our low-solubility samples (Table 1). High-solubility samples were characterised by a proportion of α-helical structures between 16 and 23% (Table 1). Five interactions govern the secondary and tertiary structure of FBPI, including covalent bonds, hydrogen, ionic and hydrophobic bonding, and electrostatic interactions [13,18]. Additionally, the proximity of a glycine residue to an asparagine residue promotes the formation of turn structures by providing extra flexibility to the protein’s main chain [47].

Temperature appears to play a critical role in governing the solubility of FBPI through its impact on protein structure and interactions (Figure 4, Figure 5 and Figure 6). Low temperature affects various interactions; for example, hydrophobic attraction is minimal below 20 °C, while hydrogen bonding and electrostatic interactions are maximised [48]. Various amino acids can form H-bonds via their side chains, especially those that contain a hydroxyl (Ser and Thr) or amide (Asn and Gln) group, or charged residues such as Lys, Arg, Asp, and Glu [49]. Faba beans are abundant in polar and hydrophilic amino acids, such as Glu, Arg, Asp, and Lys [47]. Assessment of the primary structures of faba bean legumin and vicilin, major storage proteins, reveals that they contain 10.8 and 11.2% of Ser and Thr combined, respectively. These amino acids facilitate favourable hydrophilic interactions with water molecules through hydrogen bonds and electrostatic interactions. Hence, a rise in β-sheet and α-helical confirmations has been observed as temperature and, consequently, solubility increased (Table 1). Relatively weak intramolecular hydrogen bonds may explain the lower α-helix content compared to β-sheet content. Alkaline conditions were reported to decrease β-sheet and random coil structures [50], which was not observed in the current study (Figure 4, Figure 5 and Figure 6). On the other hand, enhanced aggregation, and thus lower solubility, was depicted in a rise in inter- and intramolecular β-sheets (Table 1) and sedimentation by centrifugation, as the supernatant sample obtained at 15 °C contained fewer large aggregates remaining in the loading well (Figure 7A).

As the temperature increased, hydrophobic interactions among legumin and vicilin molecules prevailed. At the same time, hydrogen and ionic attractions were minimised, with the overall impact leading to the dissociation of these multimeric proteins into smaller subunits or monomers (Figure 7A,B). Alterations in protein structure may thus enhance the protein inter- and intramolecular repulsions, resulting in an elevation in the water solubility of FBPI [51]. For example, the absence of convicilin in the highest solubility group further corroborates the impact of temperature on altering the protein’s structural stability. Similar to pea protein isolate, elevated temperature likely disrupts the globular structure and impedes protein refolding, thereby diminishing the FBPI particle size through increased disassociation of legumin protein. It also can be reflected in the less negative zeta potential at higher temperature levels (Figure 3).

In addition to temperature, pH is another factor influencing the solubility of faba bean protein dispersions. The shift in pH influences charge distribution on proteins and cations, reduces the formation of hydrogen and ionic bonds, and minimises attractive Van der Waals forces. Like soybean isolate, some parts of faba bean protein may have unfolded during pH shift, resulting in a molten globular structure in a more alkaline environment [52]. As Figure 1 show, the solubility of FBPI was always greater at pH 7.5 than that at pH 6.8 at each temperature. At higher pH, the increased net-negative charge of protein molecules promotes electrostatic repulsion, preventing them from aggregating and thus enhancing their solubility [53]. Moreover, within the pH range of 7–8, legumin undergoes partial dissociation into a trimeric configuration. This dissociated state exhibits reduced structural organization (Figure 4, Figure 5 and Figure 6), facilitating its enhanced attachment within the interfacial region [54]. However, as temperature increases, the reduction of hydrogen bonding and enhancement of hydrophobic interactions co-occur. It engaged the polypeptide chains in both 11S and 7S globulins to transit from the stable multimeric structure to monomers and smaller molecules, inhibiting low pH’s effect on solubility. This is likely why the solubility of FBPI was similar at pH 6.8 and pH 7.5 at elevated temperatures (Figure 1).

WH was also one of the important processing parameters influencing FBPI solubility in this study (Figure 1). Solubility decreased with increasing WH levels at each temperature and pH. High levels of calcium and magnesium ions can cause complex formation of proteins in aqueous systems [55]. Approximately 15% of amino acids in legumin and vicilin are negatively charged, i.e., Asp and Glu, and can complex with divalent cations. This leads to conformational alterations in the protein structure through diverse mechanisms [2]. Calcium and magnesium in a solution can also shield the electric double-layer surrounding the protein, thereby decreasing the zeta potential and electrostatic repulsion [18]. Therefore, the increasing level of WH results in diminishing protein solubility observed in the current study (Figure 1). However, the influence of WH on protein solubility is contingent upon pH and temperature conditions (Figure 1). Elevated temperature and increasing pH have the potential to mitigate the adverse effects of water hardness on the solubility of FBPI. Notably, at a temperature of 65 °C, the impact of WH becomes negligible, resulting in a solubility of approximately 100% (Figure 1). Additionally, it has been observed that certain solubility values exceeded 100%, while an experimental error should not be excluded; this observation was previously attributed to the presence of dissolved non-protein nitrogen sources within the faba bean protein isolate [56].

Compared to distinct solubility changes under different temperatures, pH, and WH, stirring rate and time of stirring showed a minor influence, which appears to be pH-dependent. The higher stirring rate and longer time mainly contributed to increased solubility at pH 6.8. In particular, the solubility at a stirring rate of 1500 rpm was slightly greater than that at 1000 rpm at pH 6.8. However, when pH was adjusted to 7.5, the solubility at these two stirring rates appeared similar. The extent of stirring (stirring time) shared commonality with the rate. At pH 6.8, the solubility incrementally increased with time, while at pH 7.5, solubility showed an increase trend from 30 to 60 min, and then remained basically unchanged for 90 min. It appears that shear forces at these specific rates and solution conditions did not substantially affect the secondary structure of FBPI dispersions (Figure 1, Figure 2 and Figure 3). It is possible that the shear rates applied were insufficient to cause substantial structural changes in the protein, or that the protein’s structure and stability were resilient to the shear forces within the studied range.

## 5. Conclusions

The solubility of FBPI dispersions appeared contingent upon solution parameters, including temperature, pH, and water hardness. These factors impact the balance between various interactions, leading to different solubility levels. At low temperature, pH of 6.8, and greater water hardness, hydrogen and electrostatic attractions were likely enhanced, leading to greater particle size and consequently poorer solubility. Conformational changes and a rise in intramolecular β-sheet aggregates and aggregated β-sheets indicative of extensive aggregation confirmed this. The balance of interactions changes when temperature and/or pH are elevated, while the water hardness is diminished. While hydrophobic interactions are maximised at higher temperatures, the main forces may be hydrogen and ionic bonding, which are usually minimised under these conditions. This leads to particle dissociation demonstrated by smaller particle sizes and minimised inter- and intramolecular β-sheet motifs. This study demonstrated that FBPI reached the greatest solubility at pH 7.5 and 65 °C in the absence of water hardness. Nevertheless, the results of the present study provided useful insights linking protein solubility with protein reactivity and structural changes. Consequently, a greater understanding of the secondary structure of faba bean proteins and how processing conditions govern its changes may expand its utilisation in various commercial applications in the future.

## Data Availability

The original contributions presented in this study are included in the article; further inquiries can be directed to the corresponding author.
